# Association between cardiovascular health assessed by life’s essential 8 and hyperuricemia in U.S. adults: the NHANES 2009-2020

**DOI:** 10.3389/fendo.2024.1445787

**Published:** 2024-09-04

**Authors:** Meng Wang, Heyu Meng

**Affiliations:** ^1^ Department of Computer and Simulation Technology, Faculty of Health Service, Naval Medical University, Shanghai, China; ^2^ China-Japan Union Hospital of Jilin University, Changchun, China

**Keywords:** life’s essential 8, hyperuricemia, cardiovascular health, NHANES, cross sectional study

## Abstract

**Background:**

This study presented the new Life’s Essential 8 (LE8) framework for examining cardiovascular health (CVH) to analyze the potential relationship between the latter and hyperuricemia (HUA) in the U.S. population.

**Methods:**

Data on individuals aged at least 20 years were collected from the National Health and Nutrition Examination Survey (NHANES) 2009-2020. Smoothed curve fitting and multivariate logistic regression analyses were then performed on a sample of 25,681 adults to explore the association between LE8 and HUA. A sensitivity analysis was conducted to examine the robustness of the research findings.

**Results:**

The study found a strong negative association between LE8 and HUA, with an odds ratio (OR) of 0.71 and a 95% confidence interval (CI) from 0.69 to 0.73 after adjusting for multiple confounding factors. The sensitivity analysis further validated the robustness of this association. This analysis consistently showed negative associations across different genders, ages, races, and education levels (*p* < 0.05), but there were no significant relationships with marital status. The association between uric acid levels and LE8 displayed an inverted L-shaped curve, with an inflection point around 41.43.

**Conclusions:**

The findings indicate a strong negative relationship between LE8 and HUA among the U.S. population, suggesting that higher scores on the LE8, which assesses CVH, were associated with reduced uric acid levels. The consistent negative association underscores the LE8 framework’s potential as a valuable tool for understanding and managing HUA in CVH.

## Graphical Abstract

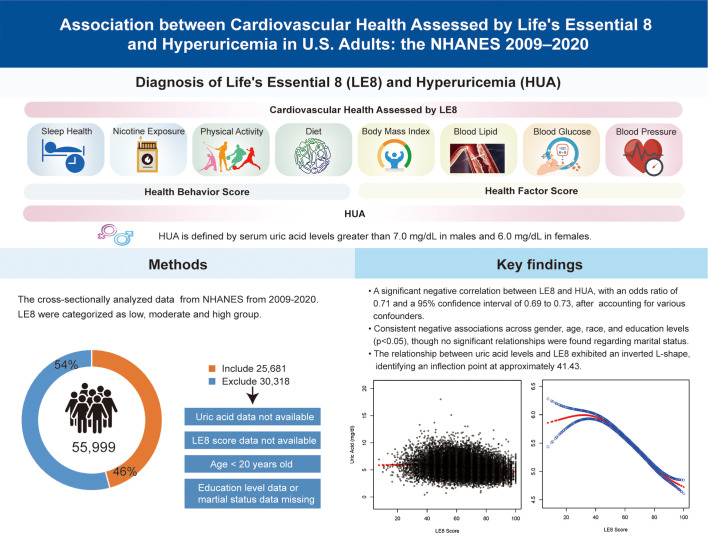

## 1 Introduction

Hyperuricemia (HUA), a well-known metabolic disorder, is primarily caused by disruptions in purine metabolism. Globally, statistics indicate that 15 to 20% of the population is affected by HUA (1), and the prevalence of this condition is rising. During the 2015–2016 period, the prevalence of HUA was 20.2% among adult males and 20.0% among adult females in the US (2). Over time, HUA has become a significant global public health concern that is strongly associated with the formation and mortality of various diseases, including gout (3), severe kidney disease (4), and elevated plasma aldosterone concentration (5). Therefore, the management and prevention of HUA are crucially important in clinical practice.

Previous studies have demonstrated a correlation between cardiovascular disease (CVD) and HUA (6, 7), with CVD being the primary cause of morbidity and mortality worldwide (8). This underscores the necessity for epidemiological research and tertiary prevention efforts. In 2022, the American Heart Association (AHA) introduced “Life’s Essential 8” (LE8), a framework comprising eight criteria to assess CVH (9). It includes a health behavior score (HBS) for diet, sleep health, physical activity, and nicotine exposure, as well as a health factor score (HFS) for blood pressure, blood glucose, lipid levels, and body mass index (BMI). The dynamic progression of CVD is widely identified, with smoking, hypertension, diabetes, and dyslipidemia being identified as primary risk factors (10). However, LE8 provides a broad analytical approach, resulting in a more precise evaluation of CVH. It also offers a comprehensive method for quantifying CVH than other single factors associated with HUA. Since its introduction, implementing the ideal CVH state as defined by LE8 has not only enhanced the prognosis for patients with CVD (11) but has also lowered the risk of stroke, diabetes, renal disease, and adult-onset asthma (12–14), among other conditions.

Some recent studies have confirmed that LE8 is associated with several metabolic diseases, such as diabetes mellitus (15) and osteoporosis (16). Moreover, as HUA is a metabolic disorder strongly associated with lifestyle factors, it may also correlate with LE8. Thus, the LE8 framework was used for this study to elucidate the influence of health behaviors on HUA. However, the association between HUA and LE8 has been underexplored, and no research has established this relationship within the U.S. adult population. These findings suggested that individuals who maintain a reasonable LE8 score may have a lower risk of developing HUA, as a higher LE8 score indicates a healthier cardiovascular system. This cross-sectional study examined adults from the U.S. population to investigate the possible association between HUA and LE8. The data for this analysis was obtained from the National Health and Nutrition Examination Survey (NHANES), which lasted from 2009 to 2020.

## 2 Material and methods

### 2.1 Data source and participant cohort

This study used data from NHANES, accessible at https://www.cdc.gov/Nchs/Nhanes/. It is a nationally representative survey conducted by the National Center for Health Statistics (NCHS) that collects comprehensive health and nutrition data from households throughout the US population. This survey included a comprehensive questionnaire, demographic, dietary, examination, and laboratory data. The NCHS Research Ethics Review Board thoroughly reviewed and approved the research methodologies of NHANES, and all participants provided written informed consent.

**Figure 1:**
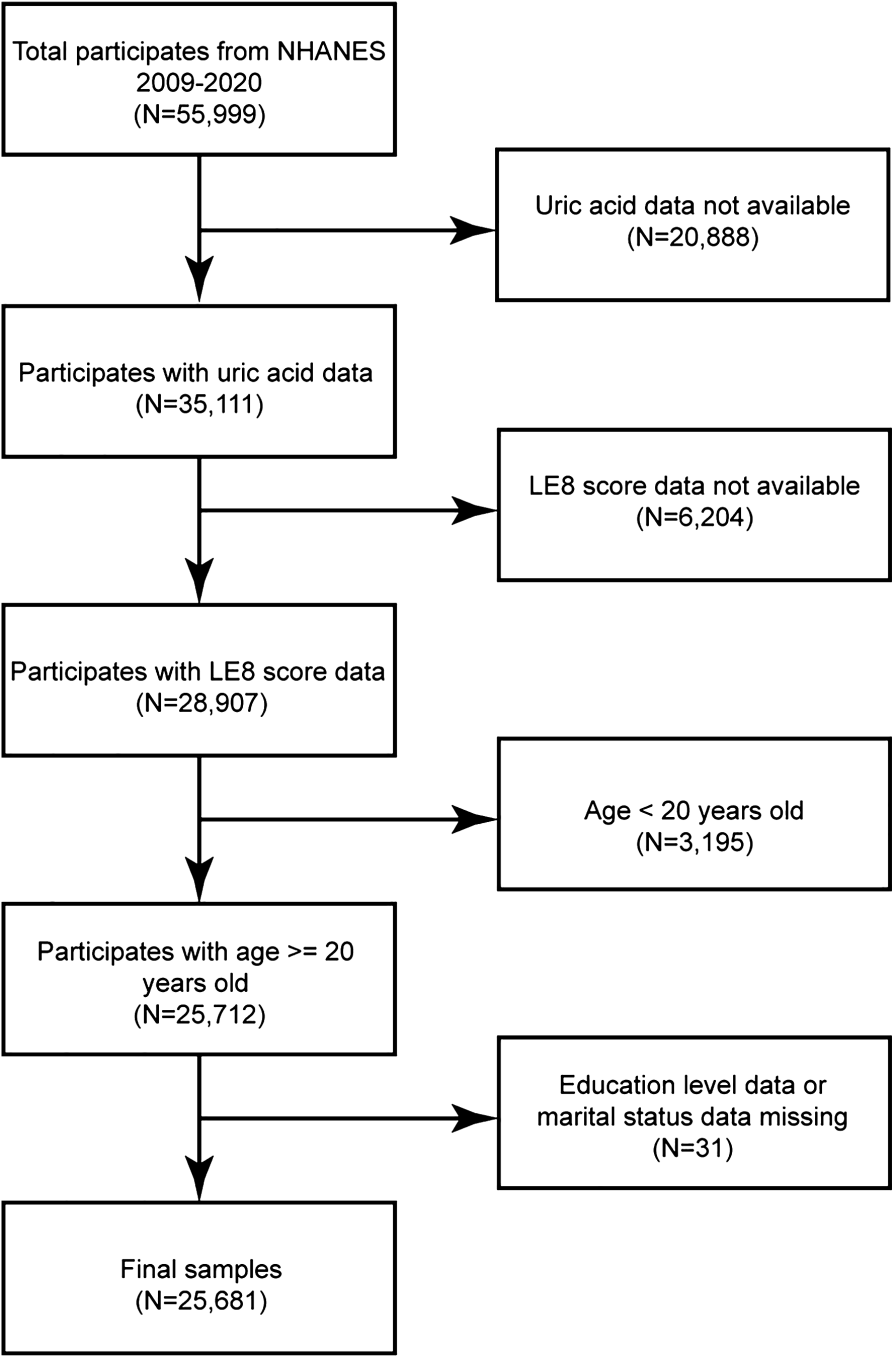
A flowchart for participant selection.

A total of 55,999 individuals were included in the NHANES 2009-2020 datasets. This study was limited to adults with LE8 scores (i.e., those without > 2 missing variables out of the 8 in the LE8 score assessment), complete data on uric acid levels, and detailed demographic information. Participants who were missing standard biochemical profile data for uric acid (N = 20,888), those with missing LE8 scores (N = 6,204), those below the age limit (N = 3,195), and participants with insufficient details on education (N = 20) or marital status (N = 11) were excluded to ensure data integrity and consistency. This study included a final cohort of 25,681 participants (**Figure 1**).

### 2.2 Diagnosis of HUA

HUA is characterized by either urate overproduction or underexcretion. In this study, HUA was quantified using serum uric acid measurements from NHANES. According to diagnostic criteria from previous studies, HUA is defined as serum uric acid levels > 7.0 mg/dL in males and 6.0 mg/dL in females (17).

### 2.3 Calculation of LE8

The LE8, introduced by AHA, quantifies CVH through the HBS and HFS. It includes eight factors: sleep, nicotine exposure, physical activity, diet, blood pressure, blood glucose, lipid levels, and BMI. The total LE8 score is calculated by combining these eight factors, each rated from 0 to 100, without assigning any weights to the scores. Moreover, AHA guidelines classify LE8 scores into three categories: low CVH (0–49), moderate CVH (50–79), and high CVH (80–100). Data on sleep, nicotine exposure, physical activity, medication usage, and health history were obtained from self-reported participant questionnaires completed by participants. Dietary information was collected through two 24-hour dietary recall interviews at the mobile screening center and analyzed using the Healthy Eating Index 2015 (18). Exercise data included the total minutes of both moderate and high-intensity activities. Sleep duration was reported in hours.

Direct measurements included BMI, calculated as weight in Kg divided by the square of height in meters, and blood pressure, reported as diastolic and systolic values in mmHg. Lipid levels were quantified from blood samples, with non-HDL cholesterol reported in mg/dL, and blood glucose levels were expressed as a percentage of glycated hemoglobin. **Supplementary Table S1** details the quantification methods for the eight factors, and **Supplementary Table S2** illustrates the calculation of LE8 scores.

### 2.4 Covariates

Based on previous research (19, 20), the current multivariate logistic regression analysis examined potential covariates affecting the association between LE8 and HUA. Given that LE8 incorporates multiple lifestyle and health-related factors, this study limited the number of covariates to avoid model overfitting (21). The final covariates selected were gender, age, race, marital status, and educational attainment.

### 2.5 Statistical analysis

The multivariate logistic regression analysis observed a linear relationship between the independent variable (LE8) and the dependent variable (HUA). The stability of this relationship was also analyzed after adjusting for various confounding factors. Moreover, LE8 and HUA were explored *via* a multivariate logistic regression analysis. A total of 3 logistic regression models were constructed, each with varying levels of confounder adjustments:

Model 1 serves as a crude model without adjusting for any covariates; Model 2 adjusts for basic demographic characteristics, including gender, age, and race, while Model 3 adjusts for all covariates, which were further adjusted for marital status and education level based on Model 2. Furthermore, in light of ongoing debates concerning the definition of HUA, a sensitivity analysis was conducted to evaluate the robustness of the results across three models with different sets of adjusted confounding factors. A nonlinear association between LE8 and HUA was identified using smoothed curve fitting techniques and a threshold effects analysis to investigate potential thresholds across intervals. Further insights were obtained through subgroup analyses *via* stratified multivariate logistic regression, considering age, marital status, education level, race, and gender. Statistical analyses were performed with R software (v4.3.3), and statistical significance was set at a two-sided as *p <* 0.05. Descriptive analyses included complex weighting; continuous variables were expressed as mean ± standard deviation (SD), while categorical ones were reported as percentages and compared using the chi-square tests.

## 3 Results

### 3.1 Basic characteristics

This study comprises 25,681 participants, divided based on whether they had HUA. **Table 1** presents the basic characteristics of the participants. In the sample, males represented 49.92%, and the average age of participants was 49.58 ± 17.50. The mean ± SD values for LE8, HBS, and HFS were 65.12± 14.75, 63.32± 20.43, and 66.70 ± 19.81, respectively. The LE8 group consisted of the following counts (%) of participants: low (3,840, 14.95%), moderate (17,389), and high (4,452, 17.34%) groups. Similarly, the HBS group’s numbers (%) were 6,064 (23.61%), 13,624 (53.05%), and 5,993 (23.34%). For the HFS group, the percentages were as follows: 5,249 (20.44%), 12,996 (50.61%), and 7,436 (28.96%). A total of 4,663 participants (18.16%) were diagnosed with HUA. Individuals with HUA were shown to have a higher possibility of being non-Hispanic Black males aged ≥ 60, with a moderate education level, and a higher frequency of being widowed or divorced relative to those without HUA. Moreover, participants without HUA generally scored higher on the LE8, HBS, and HFS scales.

**Table 1:** Basic characteristics of 25,681 participants.

Characteristics	Overall (N = 25,681)	Non-HUA (N = 21,018)	HUA (N = 4,663)	*p*-value
**Gender (%)**				<0.001
Male	12,821 (49.92)	10,217 (48.61)	2,604 (55.84)	
Female	12,860 (50.08)	10,801 (51.39)	2,059 (44.16)	
**Age (years)**	49.58 ± 17.50	48.54 ± 17.31	54.25 ± 17.63	<0.001
**Age group (%)**				<0.001
20~39	8,500 (33.10)	7,363 (35.03)	1,137 (24.38)	
40~59	8,643 (33.66)	7,218 (34.34)	1,425 (30.56)	
≥60	8,538 (33.25)	6,437 (30.63)	2,101 (45.06)	
**Race (%)**				<0.001
Mexican American	3,593 (13.99)	3,130 (14.89)	463 (9.93)	
Other Hispanic	2,664 (10.37)	2,297 (10.93)	367 (7.87)	
Non-Hispanic White	10,619 (41.35)	8,618 (41.00)	2,001 (42.91)	
Non-Hispanic Black	5,620 (21.88)	4,361 (20.75)	1,259 (27.00)	
Other Race - Including Multi-Racial	3,185 (12.40)	2,612 (12.43)	573 (12.29)	
**Education level (%)**				<0.001
Less than 9th grade	2,266 (8.82)	1,879 (8.94)	387 (8.30)	
9-11th grade (Includes 12th grade with no diploma)	3,332 (12.97)	2,714 (12.91)	618 (13.25)	
High school graduate/GED or equivalent	5,885 (22.92)	4,750 (22.60)	1,135 (24.34)	
Some college or AA degree	8,007 (31.18)	6,480 (30.83)	1,527 (32.75)	
College graduate or above	6,191 (24.11)	5,195 (24.72)	996 (21.36)	
**Marital Status (%)**				<0.001
Married	13,641 (53.12)	11,242 (53.49)	2,399 (51.45)	
Widowed	2,932 (11.42)	2,217 (10.55)	715 (15.33)	
Divorced	3,406 (13.26)	2,739 (13.03)	667 (14.30)	
Separated	633 (2.46)	523 (2.49)	110 (2.36)	
Never married	3,490 (13.59)	2,947 (14.02)	543 (11.64)	
Living with partner	1,579 (6.15)	1,350 (6.42)	229 (4.91)	
**HBS**	63.32 ± 20.43	63.58 ± 20.57	62.16 ± 19.74	<0.001
**HBS group (%)**				<0.001
Low	6,064 (23.61)	4,895 (23.29)	1,169 (25.07)	
Moderate	13,624 (53.05)	11,081 (52.72)	2,543 (54.54)	
High	5,993 (23.34)	5,042 (23.99)	951 (20.39)	
**HFS**	66.70 ± 19.81	68.96 ± 19.49	56.49 ± 17.94	<0.001
**HFS group (%)**				<0.001
Low	5,249 (20.44)	3,577 (17.02)	1,672 (35.86)	
Moderate	12,996 (50.61)	10,532 (50.11)	2,464 (52.84)	
High	7,436 (28.96)	6,909 (32.87)	527 (11.30)	
**LE8**	65.12 ± 14.75	66.54 ± 14.66	58.70 ± 13.36	<0.001
**LE8 group (%)**				<0.001
Low	3,840 (14.95)	2,697 (12.83)	1,143 (24.51)	
Moderate	17,389 (67.71)	14,150 (67.32)	3,239 (69.46)	
High	4,452 (17.34)	4,171 (19.84)	281 (6.03)	

### 3.2 Relationship between LE8 and HUA

**Table 2** illustrates the multivariate linear regression analysis, which examines the associations between LE8, its components HBS and HFS, and HUA. Collectively, it was found that LE8 and HUA were substantially and adversely correlated. The odds ratio (OR) for Model 1 (the unadjusted one) was 0.69 (95% CI: 0.67, 0.71). This negative association was consistent after adjusting for age, race, and gender (Model 2, OR = 0.71, 95% CI: 0.69, 0.73) and further adjustments for marital status and education level (Model 3, OR = 0.71, 95% CI: 0.69, 0.73). According to Model 3, the possibility of developing HUA decreases by 29% for every ten-point increment in the LE8 score. Data also revealed that those in the high and moderate LE8 groups had a lower risk of experiencing HUA than those in the low LE8 group. Specifically, the high LE8 group had 80% lower odds of having HUA (OR = 0.20, 95% CI: 0.17, 0.23), with the moderate one being related to a 43% reduction in odds (OR = 0.57, 95% CI: 0.52, 0.61). Further, enhancing scores on seven components, while accounting for one factor, was associated with an overall improvement in scores, corresponding with lower chances of developing HUA.

**Table 2:** Relationships between LE8, Its Sub-indices, and HUA.

	Model1		Model2		Model3	
	OR (95% CI)	*p*-value	OR (95% CI)	*p*-value	OR (95% CI)	*p*-value
**Every ten-point increment for LE8**	0.69 (0.67, 0.71)	<0.0001	0.71 (0.69, 0.73)	<0.0001	0.71 (0.69, 0.73)	<0.0001
LE8 group						
Low	Ref.		Ref.		Ref.	
Moderate	0.54 (0.50, 0.58)	<0.0001	0.57 (0.52, 0.61)	<0.0001	0.57 (0.52, 0.61)	<0.0001
High	0.16 (0.14, 0.18)	<0.0001	0.19 (0.17, 0.22)	<0.0001	0.20 (0.17, 0.23)	<0.0001
**Every ten-point increment for HBS**	0.97 (0.95, 0.98)	<0.0001	0.96 (0.95, 0.98)	<0.0001	0.97 (0.96, 0.99)	0.0010
HBS group						
Low	Ref.		Ref.		Ref.	
Moderate	0.96 (0.89, 1.04)	0.3107	0.96 (0.89, 1.04)	0.3548	0.99 (0.91, 1.07)	0.7451
High	0.79 (0.72, 0.87)	<0.0001	0.77 (0.70, 0.85)	<0.0001	0.81 (0.74, 0.90)	<0.0001
**Every ten-point increment for HFS**	0.72 (0.71, 0.73)	<0.0001	0.73 (0.71, 0.74)	<0.0001	0.73 (0.71, 0.74)	<0.0001
HFS group						
Low	Ref.		Ref.		Ref.	
Moderate	0.50 (0.47, 0.54)	<0.0001	0.50 (0.47, 0.54)	<0.0001	0.51 (0.47, 0.55)	<0.0001
High	0.16 (0.15, 0.18)	<0.0001	0.18 (0.16, 0.20)	<0.0001	0.18 (0.16, 0.20)	<0.0001

Model 1: Unadjusted for covariates. Model 2: Adjustments made for race, age, and gender. Model 3: Adjustments made for age, marital status, education level, race, and gender. Low groups represent scores of 0-49, moderate groups represent 50-79, and high groups represent scores of 80-100.

HBS and HFS, the sub-scores of LE8, also showed a negative association with HUA. Model 3 indicated that the possibility of having HUA decreased by 3% for each ten-point increase in the HBS (OR = 0.97, 95% CI: 0.96, 0.99). Among the HBS categories, the high group had 19% lower odds of having HUA than the low one (OR = 0.81, 95% CI: 0.74, 0.90). Simultaneously, no statistical significance (*p* = 0.7451) was found for the results of the moderate group. The possibility of having HUA was 27% lower for each ten-point increment in the HFS (OR = 0.73, 95% CI: 0.71, 0.74). The moderate group experienced a 49% reduction in their chances of having HUA compared to the low HFS group (OR = 0.51, 95% CI: 0.47, 0.55), while the high group experienced a more significant 82% decrease in odds (OR = 0.18, 95% CI: 0.16, 0.20). The sensitivity analysis (**Supplementary Table S3**) found that all three models’ results remained robust when applying the other three definitions of HUA.

### 3.3 Curve fitting and threshold effect analyses

**Figure 2:**
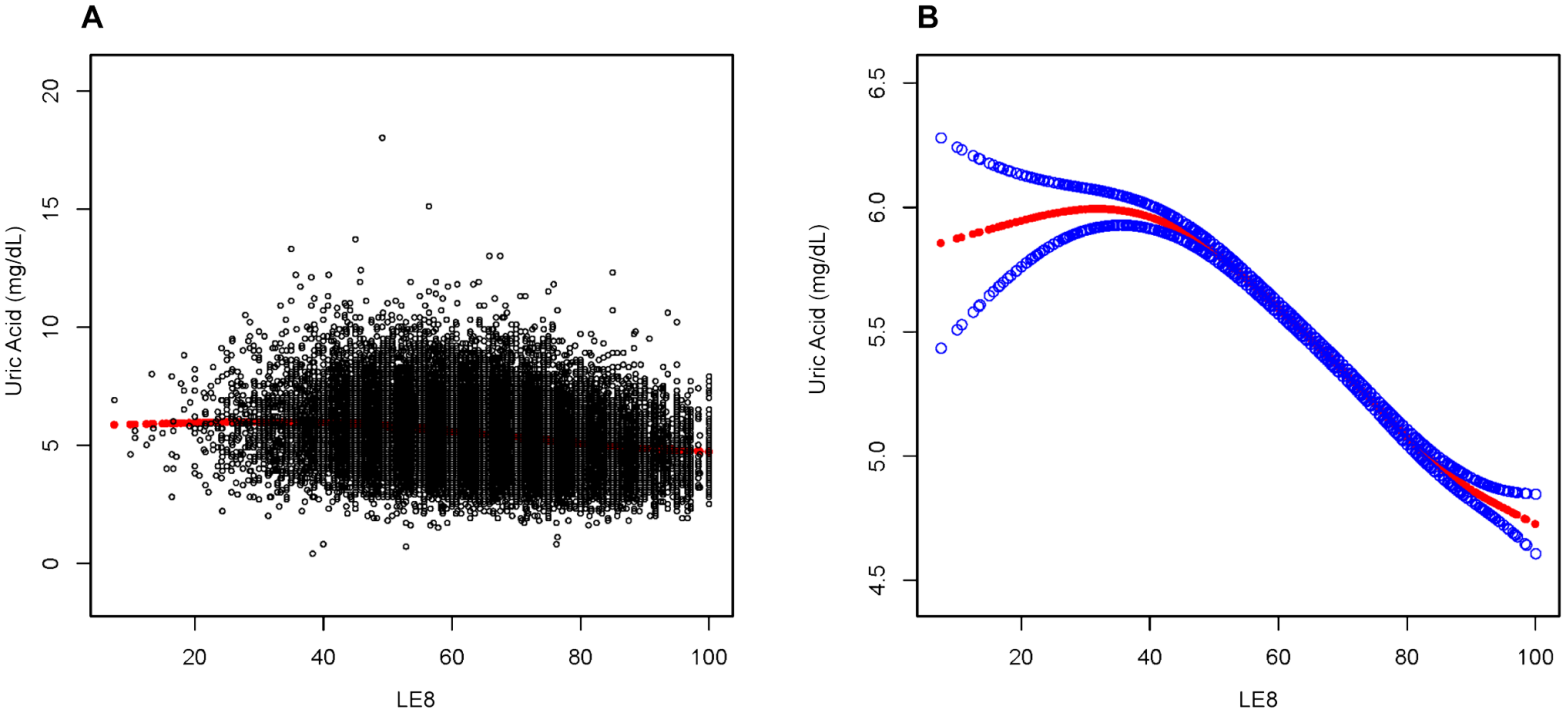
Association between LE8 and Uric Acid. **(A)** Each sample is represented by a black dot. **(B)** The smoothed curve, fitting the relationship between the two variables, is shown as a solid red line, with the 95% confidence interval for the curve indicated by the blue shaded area. This analysis has been adjusted for age, marital status, education level, race, and gender.

To further investigate the nonlinear relationship between LE8 and uric acid levels, a smoothing curve fitting was used via a linear regression model, which revealed an inverted L-shaped association between uric acid levels and LE8 scores (**Figure 2**). Moreover, a threshold effect analysis, depicted in **Table 3**, identified 41.43 points as the crucial turning point in this relationship.

**Table 3:** Analysis of the threshold effects for the association between uric acid levels and LE8 scores using linear regression.

Uric Acid(mg/dl)	Adjusted β (95% CI) P-value
Breakpoint	41.43
LE8 < Breakpoint	0.01(-0.00, 0.01) 0.1392
LE8 ≥ Breakpoint	-0.02(-0.03, -0.02) <0.0001
Log-likelihood ratio test	<0.001

Results are presented after adjusting for age, marital status, education level, race, and gender.

### 3.4 Subgroup analyses

**Figure 3:**
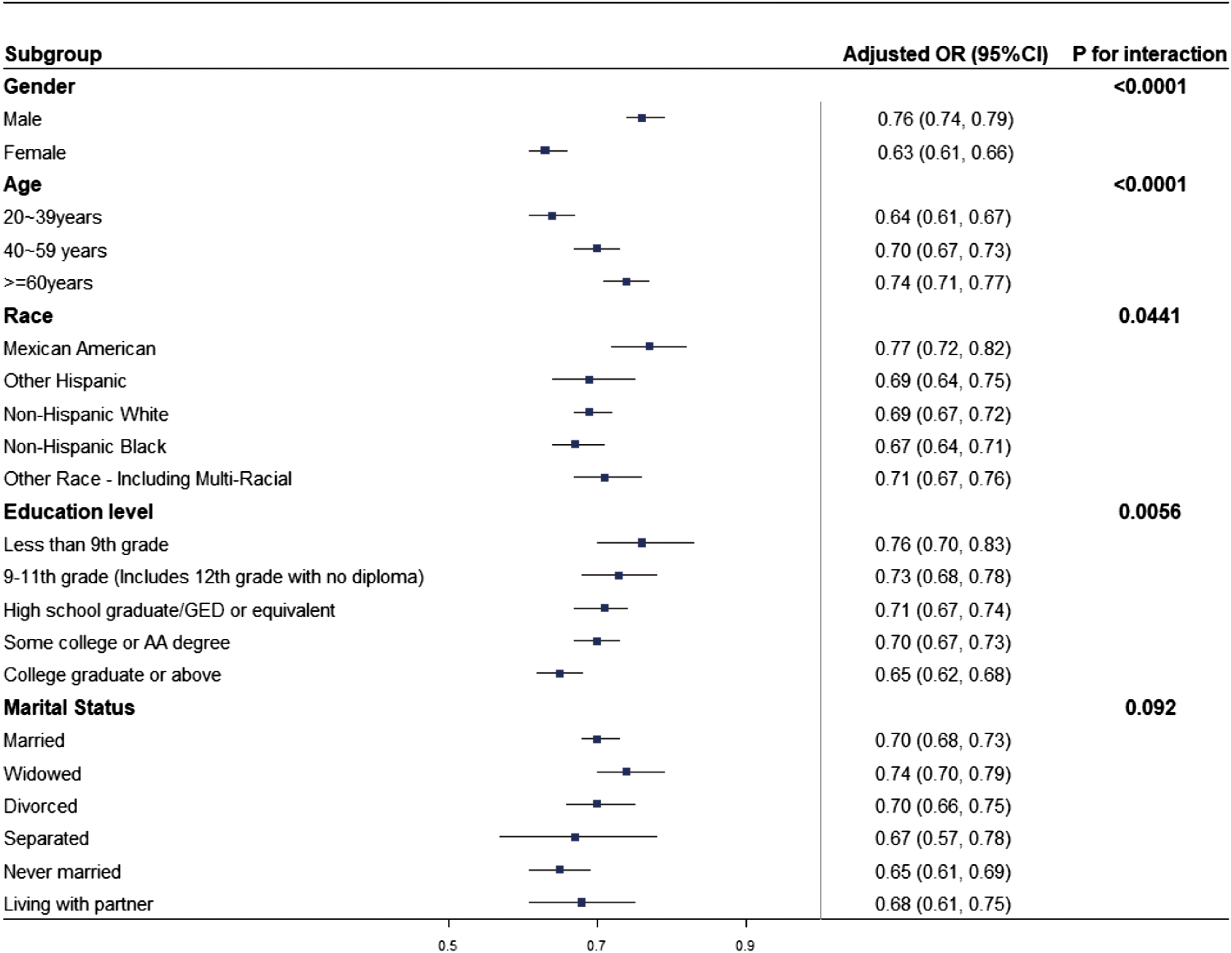
Subgroup analysis of the relationship between LE8 and HUA. The odds ratio (OR) was calculated for each 10-point increase in LE8. Each subgroup analysis was adjusted for age, marital status, education level, race, and gender.

Subgroup analyses were carried out in the study population to evaluate whether the relationship between LE8 and HUA remained consistent or differed among various demographic groups. As shown in **Figure 3**, a negative correlation between LE8 and HUA persisted across all subgroups, aligning with the findings from the overall analysis. Significant interactions (*p* < 0.05) were observed between LE8 and subgroups based on age, education level, race, and gender with HUA. Specifically, the study investigated a more pronounced negative correlation between HUA and LE8 in non-Hispanic Black females, adults aged 20 to 39, and those with a minimum college education.

### 3.5 Threshold effect analysis in Gender and Age Subgroups

Considering the significant clinical implications of age and gender differences in the association between HUA and different indicators (22–24), a comprehensive threshold effect analysis was carried out for subgroups stratified by gender and age. The inflection point for males was significantly higher than that for females. Similarly, the inflection point was higher in individuals aged 50 and older than those < 50 (**Table 4**).

**Table 4:** Threshold effect analysis of gender and age subgroups.

Uric Acid (mg/dl)	Adjusted β (95% CI) *p*-value			
	Gender		Age	
Male (N = 12,821)	Female (N = 12,860)	Age ≤ 50 (N = 12,866)	Age>50 (N = 12,815)
Breakpoint	55	40.83	43.57	50
LE8 < Breakpoint	-0.01 (-0.01, -0.00) 0.0222	-0.00 (-0.01, 0.01) 0.8873	0.00 (-0.01, 0.01) 0.4845	-0.01 (-0.01, 0.00) 0.0503
LE8 ≥ Breakpoint	-0.02 (-0.03, -0.02) <0.0001	-0.02 (-0.03, -0.02) <0.0001	-0.02 (-0.03, -0.02) <0.0001	-0.03 (-0.03, -0.02) <0.0001
Log-likelihood ratio test	<0.001	<0.001	<0.001	<0.001

For the gender subgroups, adjustments were made for age, marital status, education level, and race. Adjustments were made for the age subgroups, including marital status, education level, race, and gender.

## 4 Discussion

This cross-sectional study analyzed a nationally representative sample of 25,681 U.S. adults, revealing a strong negative correlation between HUA and LE8 scores, including its sub-components. This association remained consistent across multiple demographic subgroups, including gender, age, race, and education level, but was not significant concerning marital status. Furthermore, a nonlinear relationship between uric acid levels and LE8 scores was identified *via* smoothing curve fitting, demonstrating that higher LE8 scores were associated with lower uric acid levels. These findings underscore the importance of maintaining healthy LE8 scores through appropriate lifestyle and physical health measures to reduce the prevalence of HUA.

In previous research, a Nested Case-Control Study (25) conducted on clinical data in England indicated that gout, a metabolic disease, was related to a temporary increase in cardiovascular events. However, studies examining the relationship between LE8 and HUA are limited. Currently, only one cross-sectional study (26) has investigated this association in underdeveloped ethnic minority regions in China. This study found a negative correlation between LE8 and HUA, which aligns with the current results.

A cross-sectional analysis from the UK Biobank (27) identified a nonlinear relationship between sleep duration and HUA, with significant gender variations. Moreover, a study covering 31 provinces in China found that smoking was a specific risk factor for HUA among women (28). A cohort study also conducted in rural Henan, China, documented that regular physical activity is associated with a reduced incidence of HUA (29). A review emphasized the direct impact of diet on HUA and gout, highlighting that obesity contributes to insulin resistance (30). Multiple studies have also reported a close association between blood pressure, blood glucose levels, lipid levels, and HUA (31–35). These factors, essential determinants of health and markers of healthful lifestyle choices, are all components of LE8. Therefore, it is imperative to adopt healthy lifestyles to prevent HUA (36). Although previous studies have typically focused on individual factors, LE8 presents a holistic and practical framework for fostering healthy behaviors and attaining optimal health metrics in clinical practice (37).

The precise mechanisms associated with LE8 and HUA remain incompletely understood, however, several hypotheses have been proposed. In subgroup analyses, LE8 showed a stronger association with HUA in women (OR = 0.63, 95% CI: 0.61, 0.66), indicating that the association between CVH indicators and lower uric acid levels might be more pronounced in females. This gender difference could originate from the conversion of testosterone to estrogens (38), the quantitative dependency of HUA on the concentration of sex hormones (39), and the protective effect of female sex hormones against HUA (40). Furthermore, gender differences in cardiovascular diseases may also affect the results, especially for women who are confronted with unique risk factors (41).

Age is a crucial factor in the relationship between LE8 and HUA, with a more significant correlation observed in younger individuals aged 20-39 years (OR = 0.64, 95% CI:0.61,0.67). This observation might be explained by the increased risk of developing other prevalent conditions like osteoporosis and thyroid dysfunction with advancing age, which could obscure the relationship between LE8 and HUA (42).

Specific living environments and lifestyles may influence ethnic differences. Moreover, genetic differences among ethnic groups, particularly in genes related to urate metabolism, might enhance or impair the function of urate transporters. Thus, these genetic variations could potentially affect the regulation of serum uric acid levels (43). More importantly, the differences in cardiovascular risk factors among different ethnic groups still exist, which may also impact the relationship between LE8 and HUA (44).

A stronger correlation between the LE8 and higher educational levels (OR = 0.65, 95% CI:0.62, 0.68) could be attributed to the association between higher education and a less healthy metabolic state (45), which is also associated with reduced susceptibility to cardiovascular risk factors (46). Moreover, individuals with higher educational attainment more closely follow medical recommendations and engage in disease prevention efforts.

This study has a nationally representative and substantial sample size, which is further strengthened by the incorporation of adjustments for confounding covariates, thereby increasing the reliability of the results. Comprehensive subgroup analyses were carried out to ensure the robustness and validity of the observed correlation. However, this study has certain limitations. Due to the cross-sectional study design, it was difficult to establish a direct cause-and-effect relationship between LE8 and HUA. This limitation underscores the need for more rigorous research methodologies to unravel the complex interaction between these factors. Future longitudinal studies on a broader scale are necessary to confirm the causal association between these factors. For example, the impact of LE8 scores on HUA risk will be monitored via longitudinal studies, while interventions will evaluate the efficacy of improvements in LE8-related factors in reducing HUA. Moreover, the data may contain potential biases. For example, potential biases (selection, information, recall) may affect NHANES data due to voluntary participation excluding some populations, misreporting of personal data, and inaccurate recall of past health behaviors, impacting the accuracy of LE8 score calculations. Lastly, despite adjusting for multiple confounding factors, it is feasible that this study did not fully account for all potential confounders. To enhance the generalizability of the current findings, future studies will aim to include more diverse population samples, facilitating a more comprehensive understanding of the relationship between LE8 and HUA across different populations and thereby providing more substantial evidence for its broader applicability and effectiveness.

## 5 Conclusions

Our study demonstrates a significant inverse association between LE8 and HUA, emphasizing the importance of maintaining a healthy lifestyle and favorable health metrics in managing HUA. Future longitudinal research will explore the causal relationships between HUA and LE8 scores over time, potentially enhancing the understanding of HUA progression and management.

## Data Availability

The datasets presented in this study can be found in online repositories. The names of the repository/repositories and accession number(s) can be found below: https://www.cdc.gov/Nchs/Nhanes/.
