# Supplementary material for: Association between cardiovascular health assessed by life’s essential 8 and hyperuricemia in U.S. adults: the NHANES 2009-2020
Source: Front Endocrinol (Lausanne). 2024 Sep 4;15:1445787. doi: 10.3389/fendo.2024.1445787 (PMC11408204; doi:10.3389/fendo.2024.1445787)
Supplement: Supplementary file 1 [file Table1.docx]

Supplementary Material

**Table S1.** The quantitative method for the eight elements used in calculating the LE8 score as defined by AHA

| **Category** | **Characteristic** | **Method of measurement** | **Quantification of characteristics** | | |
| --- | --- | --- | --- | --- | --- |
| **Health behaviors** | **Diet** | Daily self-reported data on food and nutrient intake | **Metric:** Quantiles of HEI-2015 (population) | | |
|  |  |  | **Scoring (population):** | | |
|  |  |  | **Points** | **Quantile** | |
|  |  |  | 100 | ≥95^th^ percentile (top/ideal diet) | |
|  |  |  | 80 | 75^th^–94^th^ percentile | |
|  |  |  | 50 | 50^th^–74^th^ percentile | |
|  |  |  | 25 | 25^th^–49^th^ percentile | |
|  |  |  | 0 | 1^st^–24^th^ percentile (bottom/least ideal quartile) | |
|  |  |  | **Notes:** The adherence to the DASH-style diet can also be used for calculations. | | |
|  | **Physical activity** | Self-reported minutes of moderate or vigorous physical activity per week | **Metric:** Minutes of moderate intensity activity per week | | |
|  |  |  | **Scoring:** | | |
|  |  |  | **Points** | **Minutes** | |
|  |  |  | 100 | ≥150 | |
|  |  |  | 90 | ≥120 and <150 | |
|  |  |  | 80 | ≥90 and <120 | |
|  |  |  | 60 | ≥60 and <90 | |
|  |  |  | 40 | ≥30 and <60 | |
|  |  |  | 20 | ≥1 and <30 | |
|  |  |  | 0 | 0 | |
|  |  |  | **Notes:** Each minute of moderate activity should count as 1 minute and each minute of vigorous activity should count as 2 minutes toward the total for the week. | | |
|  | **Nicotine exposure** | Self-reported use of cigarettes or inhaled NDS | **Metric:** Combustible tobacco use or inhaled NDS use; or secondhand smoke exposure | | |
|  |  |  | **Scoring:** | | |
|  |  |  | **Points** | **Status** | |
|  |  |  | 100 | Never smoker | |
|  |  |  | 75 | Former smoker, quit ≥5 years | |
|  |  |  | 50 | Former smoker, quit ≥1 and <5 years | |
|  |  |  | 25 | Former smoker, quit <1 year, or currently using inhaled NDS | |
|  |  |  | 0 | Current smoker | |
|  |  |  | **Notes:** Subtract 20 points for living with an active indoor smoker in the home, except when the score is 0. | | |
|  | **Sleep health** | Self-reported average hours of sleep per night | **Metric:** Average hours of sleep per night | | |
|  |  |  | **Scoring:** | | |
|  |  |  | **Points** | **Hours** | |
|  |  |  | 100 | ≥7 and <9 | |
|  |  |  | 90 | ≥9 and <10 | |
|  |  |  | 70 | ≥6 and <7 | |
|  |  |  | 40 | ≥5 and <6, or ≥10 | |
|  |  |  | 20 | ≥4 and <5 | |
|  |  |  | 0 | <4 | |
| **Health factors** | **BMI** | Body weight (kilograms) divided by height squared (meters squared) | **Metric:** BMI (kg/m^2^) | | |
|  |  |  | **Scoring:** | | |
|  |  |  | **Points** | **Level** | |
|  |  |  | 100 | <25 | |
|  |  |  | 70 | ≥25 and <30 | |
|  |  |  | 30 | ≥30 and <35 | |
|  |  |  | 15 | ≥35 and <40 | |
|  |  |  | 0 | ≥40 | |
|  | **Blood lipids** | Plasma total and HDL cholesterol with calculation of non–HDL cholesterol | **Metric:** Non–HDL cholesterol (mg/dL) | | |
|  |  |  | **Scoring:** | | |
|  |  |  | **Points** | **Level** | |
|  |  |  | 100 | <130 | |
|  |  |  | 60 | ≥130 and <160 | |
|  |  |  | 40 | ≥160 and <190 | |
|  |  |  | 20 | ≥190 and <220 | |
|  |  |  | 0 | ≥220 | |
|  |  |  | **Note:** If the detected level is drug-treated, subtract 20 points, except when the score is 0. | | |
|  | **Blood glucose** | Casual HbA1c | **Metric:** HbA1c (%) | | |
|  |  |  | **Scoring:** | | |
|  |  |  | **Points** | **Level** | |
|  |  |  | 100 | No history of diabetes and HbA1c <5.7 | |
|  |  |  | 60 | No diabetes and HbA1c ≥5.7 and <6.4 (prediabetes) | |
|  |  |  | 40 | Diabetes with HbA1c <7.0 | |
|  |  |  | 30 | Diabetes with HbA1c ≥7.0 and <8 | |
|  |  |  | 20 | Diabetes with HbA1c ≥8 and <9 | |
|  |  |  | 10 | Diabetes with HbA1c ≥9 and <10 | |
|  |  |  | 0 | Diabetes with HbA1c ≥10 | |
|  |  |  | **Note:** If an individual patient with prediabetes is being treated to prevent the onset of diabetes and has normoglycemic levels, subtract 20 points, except when the score is 0. Furthermore, FBG can also be used for calculations. | | |
|  | **Blood Pressure** | Appropriately measured systolic and diastolic blood pressure | **Metric:** Systolic and diastolic blood pressure (mmHg) | | |
|  |  |  | **Scoring:** | | |
|  |  |  | **Points** | **Systolic** | **Diastolic** |
|  |  |  | 100 | <120 | <80 |
|  |  |  | 75 | ≥120 and <130 | <80 |
|  |  |  | 50 | ≥130 and <140 | ≥80 and <90 |
|  |  |  | 25 | ≥140 and <160 | ≥90 and <100 |
|  |  |  | 0 | ≥160 | ≥100 |
|  |  |  | **Note:** If the detected level is drug-treated, subtract 20 points, except when the score is 0. | | |

**Abbreviation:** AHA, the American Heart Association. BMI, Body Mass Index. FBG, Fasting Blood Glucose. HbA1c, Hemoglobin A1c. HEI-2015, Healthy Eating Index-2015. LE8, Life's Essential 8. NDS, nicotine-delivery system.

**Table S2.** Example Calculations of LE8

| **Metric** | **Points** |
| --- | --- |
| Diet | 80 |
| Physical activity | 80 |
| Nicotine exposure | 100 |
| Sleep health | 70 |
| BMI | 30 |
| Blood lipids | (60 - 20) = 40 |
| Blood glucose | 60 |
| Blood pressure | (50 - 20) = 30 |
| Total score | 80+80+100+70+30+40+60+30 = 490 |
| Total LE8 Score and CVH group | 490/8 = 61.25 out of 100 (Moderate CVH) |

The total LE8 score is determined by the unweighted average of eight indicators. Here is a comprehensive example showcasing the calculation of the LE8 score based on eight elements. Mr. X is a 60-year-old man who falls within the 80th percentile of the HEI-2015 diet score and engages in moderate physical activity for 45 minutes, 2 days per week. Both he and his family members refrain from smoking. Typically, he sleeps for 6 hours each night. His BMI stands at 34 kg/m2. While taking statin medication, his non-HDL-cholesterol level is 136 mg/dL. His HbA1c level is 6%. Under the control of antihypertensive medications, his average blood pressure remains at 138/85 mmHg. As a result, his LE8 score was calculated as 61.25.

**Abbreviation:** BMI, Body Mass Index. HEI-2015, Healthy Eating Index-2015. LE8, Life's Essential 8. CVH, cardiovascular health.**Table S3.** Sensitivity Analysis for Different Definitions of HUA

|  | **Model1** | | **Model2** | | **Model3** | |
| --- | --- | --- | --- | --- | --- | --- |
|  | **OR (95% CI)** | ***P*-value** | **OR (95% CI)** | ***P*-value** | **OR (95% CI)** | ***P*-value** |
| **Definition 1** |  |  |  |  |  |  |
| Every ten-point  increment for LE8 | 0.69 (0.67, 0.70) | <0.0001 | 0.70 (0.69, 0.72) | <0.0001 | 0.70 (0.69, 0.72) | <0.0001 |
| LE8 group |  |  |  |  |  |  |
| Low | Ref. |  | Ref. |  | Ref. |  |
| Moderate | 0.52 (0.48, 0.56) | <0.0001 | 0.55 (0.51, 0.59) | <0.0001 | 0.55 (0.51, 0.60) | <0.0001 |
| High | 0.16 (0.14, 0.19) | <0.0001 | 0.19 (0.17, 0.22) | <0.0001 | 0.20 (0.17, 0.22) | <0.0001 |
| Every ten-point  increment for HBS | 0.97 (0.95, 0.98) | <0.0001 | 0.96 (0.94, 0.97) | <0.0001 | 0.97 (0.95, 0.98) | <0.0001 |
| HBS group |  |  |  |  |  |  |
| Low | Ref. |  | Ref. |  | Ref. |  |
| Moderate | 0.96 (0.89, 1.03) | 0.2645 | 0.96 (0.89, 1.03) | 0.2707 | 0.98 (0.91, 1.06) | 0.6906 |
| High | 0.79 (0.72, 0.86) | <0.0001 | 0.76 (0.69, 0.83) | <0.0001 | 0.80 (0.73, 0.88) | <0.0001 |
| Every ten-point  increment for HFS | 0.72 (0.70, 0.73) | <0.0001 | 0.72 (0.71, 0.73) | <0.0001 | 0.72 (0.71, 0.73) | <0.0001 |
| HFS group |  |  |  |  |  |  |
| Low | Ref. |  | Ref. |  | Ref. |  |
| Moderate | 0.50 (0.47, 0.54) | <0.0001 | 0.51 (0.48, 0.55) | <0.0001 | 0.52 (0.48, 0.56) | <0.0001 |
| High | 0.16 (0.15, 0.18) | <0.0001 | 0.18 (0.16, 0.20) | <0.0001 | 0.18 (0.16, 0.20) | <0.0001 |
| **Definition 2** |  |  |  |  |  |  |
| Every ten-point  increment for LE8 | 0.71 (0.70, 0.73) | <0.0001 | 0.72 (0.70, 0.74) | <0.0001 | 0.72 (0.70, 0.74) | <0.0001 |
| LE8 group |  |  |  |  |  |  |
| Low | Ref. |  | Ref. |  | Ref. |  |
| Moderate | 0.61 (0.56, 0.67) | <0.0001 | 0.59 (0.54, 0.65) | <0.0001 | 0.59 (0.54, 0.65) | <0.0001 |
| High | 0.18 (0.16, 0.21) | <0.0001 | 0.22 (0.19, 0.26) | <0.0001 | 0.22 (0.19, 0.27) | <0.0001 |
| Every ten-point  increment for HBS | 0.96 (0.95, 0.98) | <0.0001 | 0.97 (0.95, 0.99) | 0.0025 | 0.98 (0.96, 1.00) | 0.0372 |
| HBS group |  |  |  |  |  |  |
| Low | Ref. |  | Ref. |  | Ref. |  |
| Moderate | 0.98 (0.90, 1.07) | 0.6200 | 0.99 (0.91, 1.09) | 0.8469 | 1.01 (0.92, 1.11) | 0.8253 |
| High | 0.78 (0.70, 0.87) | <0.0001 | 0.82 (0.73, 0.91) | 0.0004 | 0.85 (0.76, 0.96) | 0.0078 |
| Every ten-point  increment for HFS | 0.75 (0.73, 0.76) | <0.0001 | 0.74 (0.72, 0.75) | <0.0001 | 0.73 (0.72, 0.75) | <0.0001 |
| HFS group |  |  |  |  |  |  |
| Low | Ref. |  | Ref. |  | Ref. |  |
| Moderate | 0.55 (0.51, 0.60) | <0.0001 | 0.50 (0.46, 0.55) | <0.0001 | 0.51 (0.46, 0.55) | <0.0001 |
| High | 0.19 (0.17, 0.21) | <0.0001 | 0.19 (0.17, 0.22) | <0.0001 | 0.19 (0.17, 0.22) | <0.0001 |
| **Definition 3** |  |  |  |  |  |  |
| Every ten-point  increment for LE8 | 0.72 (0.70, 0.73) | <0.0001 | 0.72 (0.70, 0.74) | <0.0001 | 0.72 (0.70, 0.74) | <0.0001 |
| LE8 group |  |  |  |  |  |  |
| Low | Ref. |  | Ref. |  | Ref. |  |
| Moderate | 0.62 (0.57, 0.68) | <0.0001 | 0.59 (0.54, 0.64) | <0.0001 | 0.59 (0.54, 0.64) | <0.0001 |
| High | 0.19 (0.16, 0.22) | <0.0001 | 0.22 (0.19, 0.26) | <0.0001 | 0.22 (0.19, 0.26) | <0.0001 |
| Every ten-point  increment for HBS | 0.97 (0.95, 0.98) | <0.0001 | 0.97 (0.95, 0.99) | 0.0010 | 0.98 (0.96, 1.00) | 0.0202 |
| HBS group |  |  |  |  |  |  |
| Low | Ref. |  | Ref. |  | Ref. |  |
| Moderate | 0.98 (0.91, 1.06) | 0.6483 | 0.99 (0.91, 1.08) | 0.8056 | 1.01 (0.93, 1.10) | 0.8480 |
| High | 0.79 (0.72, 0.87) | <0.0001 | 0.82 (0.74, 0.91) | 0.0002 | 0.86 (0.77, 0.95) | 0.0044 |
| Every ten-point  increment for HFS | 0.75 (0.73, 0.76) | <0.0001 | 0.73 (0.72, 0.75) | <0.0001 | 0.73 (0.72, 0.75) | <0.0001 |
| HFS group |  |  |  |  |  |  |
| Low | Ref. |  | Ref. |  | Ref. |  |
| Moderate | 0.57 (0.52, 0.61) | <0.0001 | 0.51 (0.47, 0.55) | <0.0001 | 0.51 (0.47, 0.55) | <0.0001 |
| High | 0.20 (0.18, 0.22) | <0.0001 | 0.20 (0.17, 0.22) | <0.0001 | 0.19 (0.17, 0.22) | <0.0001 |

Definition 1(1): Defined HUA as SUA levels exceeding 7.0 mg/dL in male adults and 5.7 mg/dL in female adults. Definition 2(2): Defined HUA as SUA levels above 7.0 mg/dL, irrespective of gender. Definition 3(3): Defined HUA as SUA levels greater than 6.8 mg/dL.

Model 1: Unadjusted for covariates. Model 2: Adjustments made for race, age, and gender. Model 3: Adjustments made for age, marital status, education level, race, and gender. Low groups represent scores of 0-49, moderate groups represent scores of 50-79, and high groups represent scores of 80- 100.

**Reference**

1. Wang K, Wu J, Deng M, Nie J, Tao F, Li Q, et al. Associations of Oxidative Balance Score with Hyperuricemia and Gout among American Adults: A Population-Based Study. *Front Endocrinol (Lausanne)* (2024) 15:1354704. Epub 20240626. doi: 10.3389/fendo.2024.1354704.

2. Jing Y, Ma L, Zhang Y, Li X, Jiang J, Long J, et al. Impact of Health Literacy, Social Support, and Socioeconomic Position on the Serum Uric Acid Level in Asymptomatic Hyperuricaemia Patients in China: A Structural Equation Model. *BMC Public Health* (2024) 24(1):1606. Epub 20240617. doi: 10.1186/s12889-024-19085-6.

3. Liu X, Chen TY, Gao TY, Shi KQ, Yin FQ, Yu YX, et al. Pro-Inflammatory Diets Promote the Formation of Hyperuricemia. *Front Endocrinol (Lausanne)* (2024) 15:1398917. Epub 20240621. doi: 10.3389/fendo.2024.1398917.
